# Supplementary material for: Automatic analysis of treadmill running to estimate times to fatigue and exhaustion in rodents
Source: PeerJ. 2018 Jul 6;6:e5017. doi: 10.7717/peerj.5017 (PMC6037152; doi:10.7717/peerj.5017)
Supplement: Supplemental Information 2 — One page instruction on steps to calculate times to fatigue and exhaustion using Excel template. [file peerj-06-5017-s002.pdf]

Calculation of Times to fatigue and exhaustion using Excel template

1) Create two columns with the data in a separate spreadsheet:
   first column – time;
   second column – distance from back.
2) Select these two columns and copy-paste them to the A and B columns of template.
3) Check that beginning and finish of the record correspond to what you expect (Time min and Time max)
4) Calculate Time Conversion from the videofile (see the manual). TimeConversion for analysis of videofeed is 1.
5) Select the step (length of averaging interval, usually 1 min).
6) Select the interval to be analyzed (Start and Finish). Pressing "Copy times" button copies Time min and Time max into Start and Finish field.

7) Press the button "Calculate TFE" (TFE – Time to Fatigue and Exhaustion).

8) Inspect calculated results and graphs.

9) If any parameter in the column E (TimeConversion, Step, or times for Start and Finish) needs to be changed, do it, and go to step (7) – press "Calculate TFE" button.
10) If only parameters in columns M and O need adjustments, results update automatically after the change.

11) Check and adjust Upper and Lower border (position as 1 and 0) for normalization.

12) Check and adjust thresholds for Fatigue and Exhaustion, and the number of steps to consider that threshold was passed for each parameter.

Times to fatigue and exhaustion are calculated.

If the beginning of the analyzed interval is not the beginning of the recording (Start > Time min), the times to fatigue and exhaustion relative to the beginning of recording will be also shown.
