# Supplementary material for: Automatic analysis of treadmill running to estimate times to fatigue and exhaustion in rodents
Source: PeerJ. 2018 Jul 6;6:e5017. doi: 10.7717/peerj.5017 (PMC6037152; doi:10.7717/peerj.5017)
Supplement: Supplemental Information 3 — Complete description of the use of Excel template. [file peerj-06-5017-s003.pdf]

Author's statement

Author gives the permission to use the template and modify the code (Free Open Source License). However, it is expected that publications which use this template will contain the reference to the manuscript, which includes this template as the Supplemental material.

Introduction

This template does basic processing of a string of data pairs (time; position). The length is limited only by Excel limitations: typical video tracking analysis makes 3-8 points per second, thus providing 10000-50000 points per hour. In some cases a rat can run longer than an hour. The number of points per second is not fixed or stable, therefore it requires some software to average it using fixed time intervals. Usually, time interval is one minute, because it is the easiest to implement with human observer. However, in some cases shorter or longer intervals can be utilized. In fact, when we established treadmill running paradigm in our lab, we looked if alternative length will provide more information. This could be the case for experiments with relatively short running, like MaxVO2 protocols, when the speed of belt is increased over time, and whole length of running session is around 15-20 minutes. Alternatively, if the rat runs longer than 60 minutes, it is easier to have less than 60 points for the presentation.

This template is not fool-proof. It assumes that data are arranged properly. The time is supposed to increase with increasing line number. Reversing the time sequence will not by itself cause an error in execution, but may cause erroneous results. To make processing and analysis easy, the author prefers to identify the beginning of the run as time 0 when the videotracking is initiated.

Date/time format in the Excel includes the date (see the description below). When the date is changed, the time changes from 11:59 to 00:00. To make data continuous during the change of date, the time needs to include the date. If done that way, the change of date will be processed properly.

Processing of data consists of three steps:

1) Preparation for the analysis; Pasting prepared data into the template. Software automatically detects the time of start and finish. Select the interval to be analyzed.
2) The analysis itself;
3) Adjustment of analysis parameters to produce results.

1. Preparation for the analysis

Prepare the data in a separate Excel spreadsheet. Most likely, the user needs to import original text file, generated by video tracking software into the Excel (**[File: Open]**; and properly assigning data types in columns).

The column with time should be in Excel time format. Excel stores times as a decimal portion of a day, e.g. 1am = 1/24 ≈ 0.041667. Whole number is the number of days passed since January 0, 1900. If you had used the time as timestamp starting with the beginning of the run at t=0, then 1 sec (shown as 0:00:01) = 0.0000116; 1 min (shown as 0:01:00) ≈ 0.0006944; etc. For simplicity of presentation, the columns may be shown as numbers (**[Format Cells: Numbers]**) or as time (**[Format Cells: Time])**. Custom format may help **[Format Cells: Custom: hh:mm:ss.0]** to show parts of second reflecting few positions which are recorded every second). Independent of the presentation format, the value will be processed as a number.

After pasting, the template automatically identifies the beginning and the end of the time.

Pasting prepared data into the template. Software automatically detects the time of start and finish (Fig.1, #1), which are minimal and maximal value of time in the column. Next, type the interval for averaging in minutes (named Step, Fig.1, #2). Usually, the step is set at 1 minute, but it can be more or less.

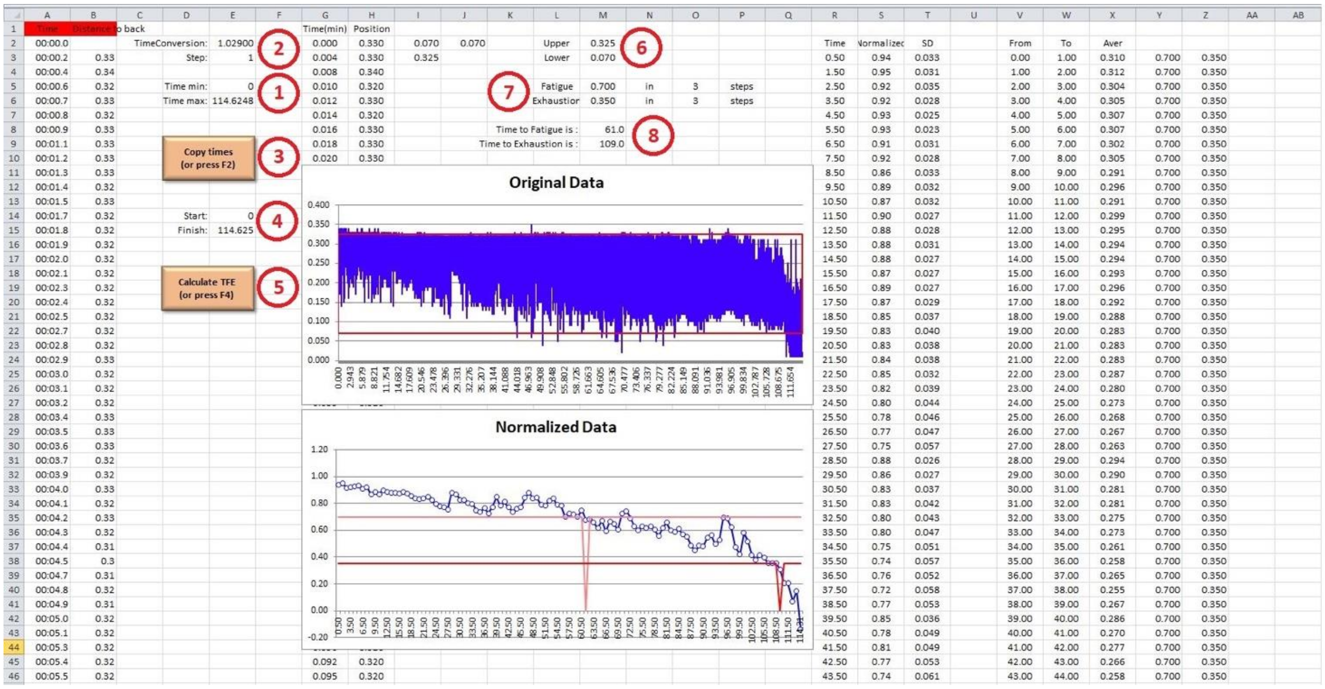

Figure 1. Screenshot of the Excel spreadsheet to calculate times of fatigue and exhaustion using results of videotracking. Circled numbers are added for referencing in the text.

Finally, correction of time should be made (TimeConversion, Fig.1, #2). TimeConversion is a coefficient between the speed of actual time and the speed of time during video file playback. Fig.2 demonstrates how this coefficient can be calculated. When the animal starts running, the time in the file is “00:00:10”, while actual embedded time stamp shows “1:10:47”. Ninety minutes later of video playback, the time in the file is “1:30:10”, while actual time stamp shows “2:43:22”. Playback time lapsed 5400 seconds, while actual time lapsed 5555 seconds. TimeConversion = 5555/5400 = 1.0287.

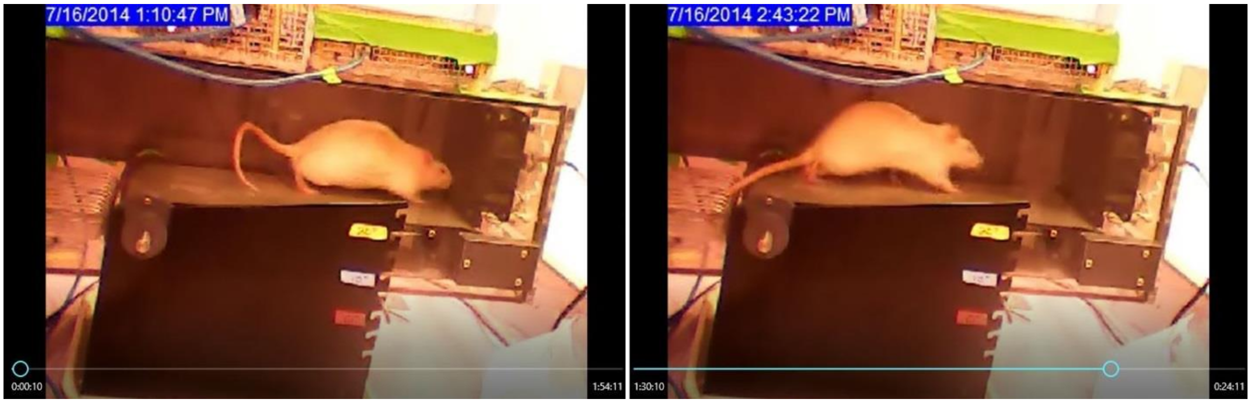

Figure 2. Screenshots of the recorded video with running rat in the beginning of exercise (left) and at the end of the run (right). See instructions how to calculate Time Conversion value.

2. Analysis

Select the interval to be analyzed. It can be done manually (Fig.1, #4). Alternatively, the complete range from minimum to maximum may be selected by pressing the button *"Copy times"* (Fig.1, #3). In the typical video tracking recording, the beginning time is usually zero, but the last number can have multiple digits after the comma. Using *"Copy time"* button simplifies recovery of initial settings to re-analyze dataset if analysis gone wrong.

The beginning of the range will serve as 0.

When initial settings are prepared, press the button *"Calculate TFE"* (TFE – Times to Fatigue and Exhaustion, Fig.1, #5).

Columns A to F are filled by the user. Columns to the right of G are filled by software, after deleting the original content of these columns.

3. Adjustment of parameters for analysis

After pressing *"Calculate TFE"* button, Excel deletes columns to the right of Column G to create space for processed data and graphs. Then, Excel subtracts minimal value at the beginning of selected interval from times in the Column A and fills the Column G with the times in minutes. Column H is filled with values of distance; technically, it is a copy of Column B.

Columns I and J serve the graph of original data, and are needed for the creation of red rectangle.

In this template, normalization is supposed to be done mostly manually. The algorithm initially provides some estimates of upper and lower level corresponding to 1 and 0. To exclude outliers we used 1% percentile on both sides (there is a built-in function in VBA, so if you would like to change it, it is just one number). In the interface, the upper and lower levels are shown in the cells M2 and M3 (Fig.1, #6).

Changing the levels automatically changes graphs, the results of calculations, and estimated time to fatigue and exhaustion.

Time to fatigue is defined as the earliest time when average position during time interval falls below threshold and does not recover in predefined number of steps. For example, threshold in the example (Fig.1, #7) is set at 0.7, and average normalized position needs to be below 0.7 for at least 3 minutes (step is 1 min).

Time to exhaustion is defined as the earliest time when average position during time interval falls below threshold and does not recover in predefined number of steps. For example, threshold in the example is set at 0.35, and average normalized position needs to be below 0.35 during at least 3 minutes (step is 1 min).

- Please, consider the step, when setting the time. It is the number measured in steps, not minutes.

Finally, time to fatigue and exhaustion is calculated automatically using settings which are described above. The number is in minutes, and is relative to the beginning of the recording. If selected interval (red box on the original graph) starts not at 0, then absolute time will also be shown where the circled label “8” is currently shown (Fig.1, #8). Calculated times are shown on the lower graph by dropping line.

If either the time to fatigue, or the time to exhaustion fails to be calculated (in case, animal never drops below one of thresholds), this time will be identified as “N/A”, and corresponding line will remain without the drop to X axis.

Columns R, S, and T contain information on averaged values of normalized position and standard deviation of position over the interval. Time is set corresponding to the middle of the interval.

Columns V, W, and X are used for calculations:

V – beginning of the interval;

W – end of the interval;

X – average absolute position over the interval (used to calculate normalized position);

Columns Y and Z are used to draw thresholds for fatigue and exhaustion, and mark the time to fatigue and the time to exhaustion.
